# Determining sensitivity and specificity of HER2 testing in breast cancer using a tissue micro-array approach

**DOI:** 10.1186/bcr3208

**Published:** 2012-06-13

**Authors:** Tim JA Dekker, Susan Ter Borg, Gerrit KJ Hooijer, Sybren L Meijer, Jelle Wesseling, James E Boers, Ed Schuuring, Jos Bart, Joost van Gorp, Wilma E Mesker, Judith R Kroep, Vincent THBM Smit, Marc J van de Vijver

**Affiliations:** 1 Department of Medical Oncology, Leiden University Medical Center, Albinusdreef 2, Leiden, 2333 ZA, The Netherlands; 2 Department of Surgery, Leiden University Medical Center, Albinusdreef 2, Leiden, 2333 ZA, The Netherlands; 3 Department of Pathology, Academic Medical Center, Meibergdreef 9, Amsterdam, 1100 DD, The Netherlands; 4 Department of Pathology, Netherlands Cancer Institute, Plesmanlaan 121, Amsterdam, 1066 CX, The Netherlands; 5 Department of Pathology, Isala Klinieken, Stilobadstraat 3, Zwolle, 8021 AB, The Netherlands; 6 Department of Pathology, University Medical Center Groningen, Hanzeplein 1, Groningen, 9713 GZ, The Netherlands; 7 Department of Pathology, Diakonessenhuis, Bosboomstraat 1, Utrecht, 3582 KE, The Netherlands; 8 Department of Pathology, Leiden University Medical Center, Albinusdreef 2, Leiden, 2333 ZA, The Netherlands

## Abstract

**Introduction:**

Overexpression of the human epidermal growth factor receptor 2 (HER2) as a result of HER2 gene amplification is associated with a relatively poor prognosis in breast cancer and is predictive of HER2-targeting therapy response. False-positive rates of up to 20% for HER2 testing have been described. HER2-testing laboratories are therefore encouraged to participate in external quality control schemes in order to improve HER2-testing standardization.

**Methods:**

This study investigated the feasibility of retesting large numbers of invasive breast cancers for HER2 status on tissue micro-array (TMA) as part of a quality control scheme. For this assessment different HER2 testing methods were used including HER2 detecting antibodies SP3, 4B5, Herceptest and mono color silver *in situ *hybridization (SISH) and dual color SISH. Final HER2 status for each tumor on the TMA was compared to the local testing result for the same tumor. Discordances between these two results were investigated further by staining whole tumor sections.

**Results:**

For this study, 1,210 invasive breast carcinomas of patients treated in six hospitals between 2006 and 2008 were evaluated. Results from the three immunohistochemistry (IHC) and two *in situ *hybridization (ISH) assays performed on the TMAs were compared. The final HER2 status on TMA was determined with SP3, 4B5 and mono color SISH. Concordance between local HER2 test results and TMA retesting was 98.0%. Discordant results between local and TMA retesting were found in 20 tumors (2.0%). False positive HER2 IHC results were identified in 13 (1.3%) tumors; false negative IHC results in seven (0.7%) tumors.

**Conclusions:**

Retesting large volumes of HER2 classified breast carcinomas was found to be feasible and can be reliably performed by staining TMAs with SP3, 4B5 and mono color SISH in combination with full-sized slides for discordant cases. The frequency of false-positive results was lower than previously reported in the literature. This method is now offered to other HER2-testing laboratories.

## Introduction

Human epidermal growth factor receptor 2 (HER2) is a member of the family of tyrosine kinase receptors. Overexpression of the HER2 receptor generally results from HER2 gene amplification and occurs in approximately 10% to 20% of primary breast carcinomas [1,2]. Positive HER2 status of primary breast cancer has been associated with relatively poor prognosis [3] and some studies have also shown that HER2 positive tumors differ from HER2 negative tumors in their response to systemic hormonal therapy [4] and chemotherapy [5,6]. Therapy with the monoclonal antibody trastuzumab targets the extra-cellular domain of the HER2 protein, leading to receptor internalization and antibody dependent cellular cytotoxicity [7,8]. Treatment with trastuzumab was first shown to prolong survival in patients with HER2 positive metastatic breast cancer, especially when combined with chemotherapy [9]. Adding trastuzumab to adjuvant chemotherapy of patients with HER2 positive breast cancer was shown to improve patient survival and reduce the chance of developing distant metastases [10,11]. Lapatinib is an intracellular HER2 tyrosine kinase inhibitor which has been approved for trastuzumab-resistant HER2 positive metastatic breast cancer [12,13]. As a result of these clinical findings, it has become routine practice to test all invasive breast carcinomas for HER2 status. HER2 testing should be carried out in such a way that false positive and false negative test results are avoided in order to select the proper patients for HER2 targeted therapies.

The HER2 status of a tumor can be assessed by various methods, several of which have been approved for clinical use, including immunohistochemistry (IHC), FISH, SISH and CISH (fluorescence, silver and chromogenic *in situ *hybridization). A 2007 report by an American Society of Clinical Oncology/College of American Pathologists (ASCO/CAP) panel has estimated that 20% of HER2 testing might be incorrect [14]. The panel included several recommendations for improving HER2 testing variability and recommended that HER2-testing laboratories show at least 95% concordance with validated HER2 negative and positive cases. Unfortunately, published HER2 series have often found a significant number of discordant results. Paik *et al*. retested tumors treated with trastuzumab in the National Surgical Adjuvant Breast and Bowel Project protocol 31 (NSABP-31) trial which compared the addition of trastuzumab to adjuvant chemotherapy [15]. Eligibility for this trial was based on local HER2 test results, but it was estimated that 18% of tumors were tested as false positive due to inaccurate test and/or interpretation methods. Central retesting of tumors locally tested as HER2 positive from patients who participated in the N9831 trial identified only 85.8% of tumors as HER2 amplified [16]. The same study by Perez *et al*. also showed that the concordance between local and central testing was found for FISH (88.1%), Herceptest (81.6%) or other IHC methods (75.0%). Fewer studies have described the frequency of false-negative HER2 test results. O'Malley *et al*. reported concordant results between 94.8% to 100% for IHC and 98.5% for FISH for all HER2 negative tumors [17]. These reported inaccurate results are explained by different protocols used in HER2 testing facilities. Factors that affect test results include warm/cold ischemic time of tissue, duration of fixation, used fixative, method for antigen retrieval, antibody and test interpretation. In order to improve the reliability and standardization of HER2 results, laboratories are encouraged to participate in external quality controls in order to improve the standardization of HER2 testing. The study described here was conducted to develop a rapid and reliable method for the determination of false positive and false negative HER2-testing rates in different pathology laboratories. For this purpose, tissue blocks of HER2-tested breast cancers were collected from six different pathology laboratories and were used to create tissue micro arrays (TMAs). Because this was the first TMA assessment, different HER2 testing methods were used. Results from these methods were compared in order to determine which methods should be used for this and future TMA assessments. The final TMA testing result for each tumor was compared to the local testing result to determine the reliability of the local HER2 methods for each participating laboratory.

## Materials and methods

### TMA construction and IHC

Paraffin blocks from invasive primary breast carcinomas diagnosed in 2008 were collected from the following hospitals: Academic Medical Center (Amsterdam), Netherlands Cancer Institute (Amsterdam), Diakonessenhuis (Utrecht), Isala Klinieken (Zwolle), Leiden University Medical Center (Leiden) and University Medical Center (Groningen). Tumors from the Academic Medical Center Amsterdam were from patients treated in 2006 and 2007. Patients from the Leiden University Medical Center were treated between 2006 and 2008. Tissue blocks that were used in this study were all acquired during routine patient care. According to Dutch law, these can be freely used after anonymizing the tissues, provided these are handled according to national ethical guidelines ('Code for Proper Secondary Use of Human Tissue', Dutch Federation of Medical Scientific Societies). An H & E stained section from each tumor was used to identify an area with invasive breast cancer. From each tumor three cores with thickness of 0.6 mm were collected using the Beecher TMA instrument and inserted in a donor block. Each donor block was stained with the antibodies SP3 (Labvision, using Labvision autostainer, Fremont, CA, United States), 4B5 (Ventana medical systems, using the Benchmark XT, Tucson, AZ, United States) and Herceptest (DAKO, using Autostainer Link 48, DAKO, Glostrup, Denmark). Mono color and dual color SISH was performed with the SISH kit obtained from Ventana using the Benchmark XT.

### HER2 evaluation on TMA

**Figure 1:**
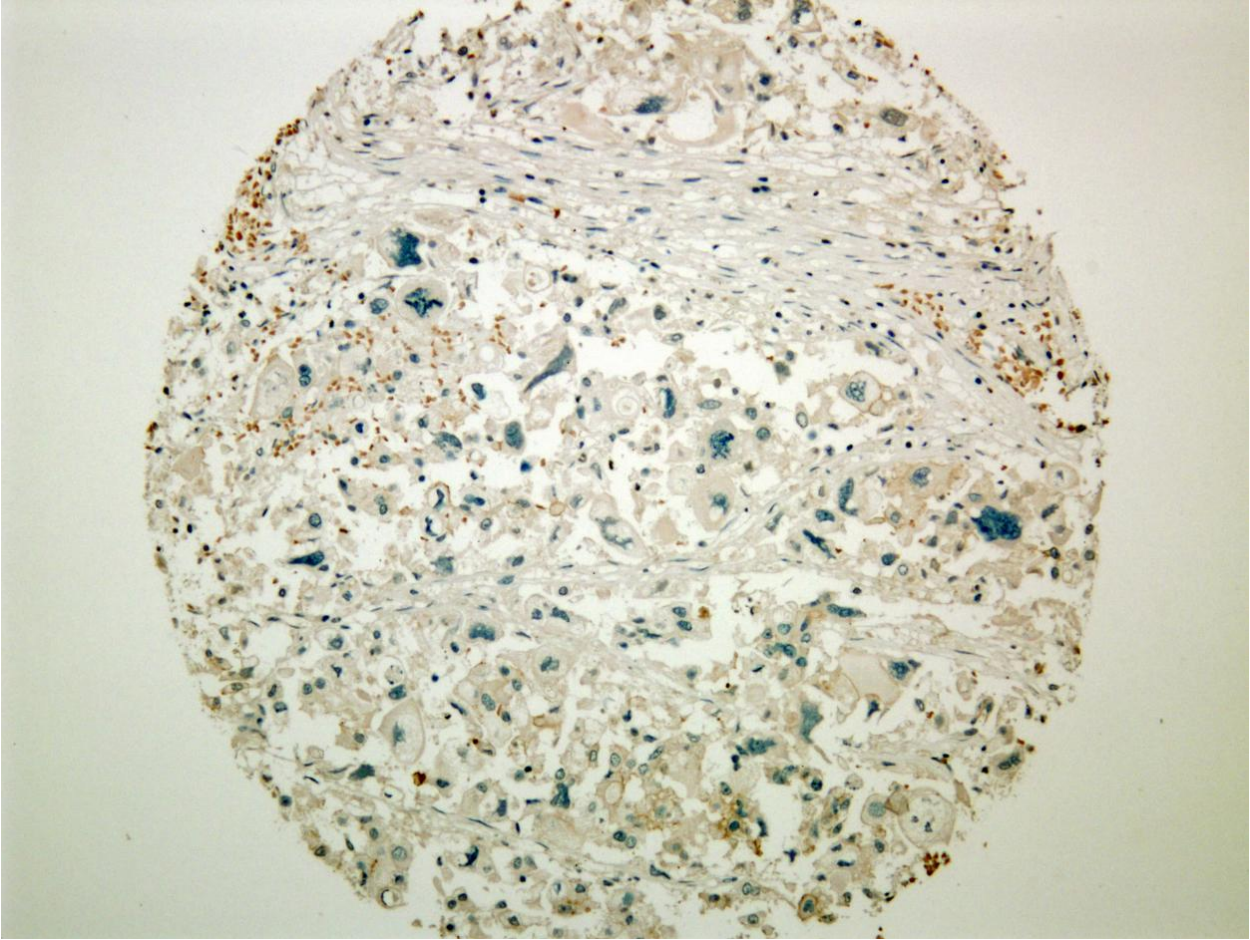
**TMA core displaying completely negative staining for HER2 (4B5 antibody)**. HER2, human epidermal growth factor receptor 2; TMA, tissue micro-array.

**Figure 2:**
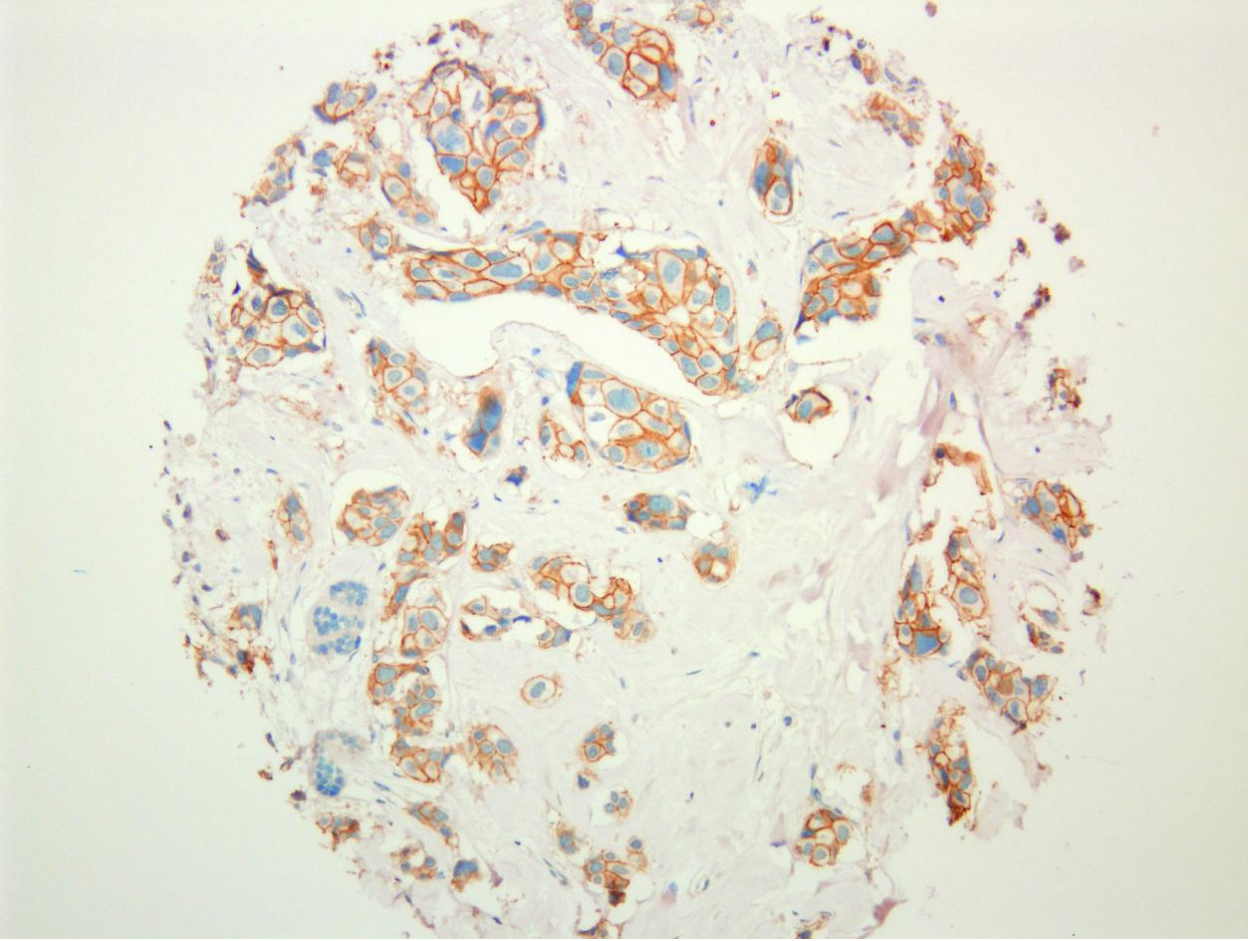
**TMA core displaying weak membranous HER2 staining (2+, 4B5 antibody)**. HER2, human epidermal growth factor receptor 2; TMA, tissue micro-array.

**Figure 3:**
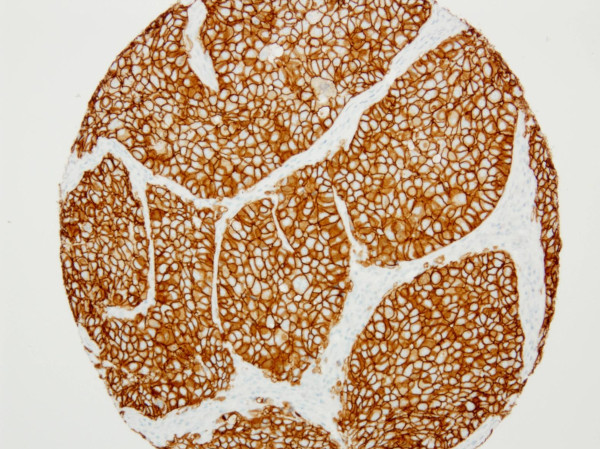
**TMA core displaying strong membranous HER2 staining (3+, 4B5 antibody)**. HER2, human epidermal growth factor receptor 2; TMA, tissue micro-array.

Scoring for IHC and *in situ *hybridization was performed according to ASCO guidelines [14]. In brief, HER2 IHC was scored as 0 when no tumor cells showed positive HER2 membrane staining, 1+ scoring represented weak partial staining of tumor cells (Figure 1), 2+ represented weak to moderate intensity membranous staining of the tumor cells (Figure 2) and 3+ staining represented strong circumferential staining of the tumor cells (Figure 3). For mono color SISH, the number of nuclear spots was counted in 20 adjacent tumor cells. If the average number of HER2 signals was six or more, the tumor was scored as HER2 amplified and if the HER2 copy number was < 6, the tumor was scored as HER2 non-amplified. For dual color SISH, the number of spots in 20 adjacent cells was counted for HER2 signals and chromosome 17 signals. When the ratio was < 1.8, the tumor was scored as HER2 non-amplified. HER2 amplification was seen when the HER2 to chromosome 17 ratio exceeded 2.2. When the HER2 to chromosome 17 ratio was between 1.8 and 2.2, tumors were considered equivocal. For the dual color SISH, the number of nuclear copies of HER2 and chromosome 17 were separately recorded as well. All three tumor cores were scored separately. In case of discordance between the cores, the highest score was used. When cores were missing due to folding of material or loss of material during the procedure, the highest scores from the remaining core(s) were considered.

### Data processing

Each TMA was scored by two pathologists. For 4B5, 51 (4.7%) out of 1,093 results showed a discrepancy between two observers (Cohen's κ = 0.787). For SP3, 37 (3.4%) from 1,077 cases showed a discrepancy (κ = 0.833). For Herceptest, 53 (4.8%) out of 1,107 cases showed a discrepancy (κ = 0.743). For 786 mono color SISH cases, 22 results (2.8%) were discordant between two observers (κ = 0.838). For 914 dual color SISH cases, 43 results (4.7%) were discordant between two observers (κ = 0.671). Significantly discrepant scores between the two observers were reviewed by one observer (TD) to resolve the final score. In order to assess the concordance between mono color and dual color SISH, TMA results from all mono color and dual color SISH tested tumors were compared. Tumors that were equivocal on dual color SISH were not considered discordant with either HER2 non-amplified and HER2 amplified mono color SISH results for the same tumor. All tumors that were discordant between mono and dual color SISH were reviewed and scored again on the TMA. When discordant results existed between mono color and dual color SISH, the IHC results from these discordant tumors were evaluated. Results of the different HER2-antibodies were evaluated by determining the number of cases with discordant results between IHC and mono color SISH: HER2 amplified tumors with 0 or 1+ scores on IHC ('false negative IHC') and HER2 non-amplified tumors with 3+ IHC scores ('false positive IHC'). Positive predictive values were calculated as the percentage of the total number of SISH amplified cases for all 3+ IHC scores.

### Comparison of HER2 score on the TMA and archival HER2 score

HER2 scores were retrieved from the pathology reports supplied by participating centers. Four centers performed HER2 testing on the surgical specimens. The other two centers routinely performed HER2 testing on the pre-operative core needle biopsies (CNB). For almost all cases, the algorithm used to obtain a HER2 score was to perform IHC staining first. When 0 or 1+ staining results were observed, the tumor was regarded as HER2 negative. A HER2 3+ score resulted in a HER2 positive score. However, the two centers that determined HER2 status on CNB also performed *in situ *hybridization in case of a 3+ result. For all other centers, *in situ *hybridization was performed only in the case of a 2+ result. If HER2 gene amplification was present, the HER2 status was scored as positive. If no HER2 gene amplification was detected, the HER2 status was scored as negative. The final HER2 score on the TMA and the HER2 scores from the report were compared for all tumors. If there was a discrepancy in the HER2 score between the TMA score and the score recorded in the pathology report, a whole tissue block of the breast carcinoma was sectioned and used to perform additional staining and *in situ *hybridization.

## Results

### Concordance between mono color and dual color SISH

A total of 1,210 invasive primary breast carcinomas were included in this study. Complete mono color SISH and dual color SISH scores were obtained for 971 tumors. The remaining 239 tumors had incomplete results, due to folding of the core, loss of tumor material or insufficient amounts of invasive breast cancer for scoring. Using mono color SISH, 881 tumors (91%) were non-amplified (HER2 copy number < 6) and 90 (9%) tumors were amplified (HER2 copy numbers > 6). For dual color SISH, 833 tumors (86%) were non-amplified (HER2 to chromosome 17 probe ratio < 1.8), 20 tumors (2%) were considered equivocal for amplification (1.8 < HER2 to chromosome 17 probe ratio < 2.2) and 118 (12%) tumors were amplified (HER2 to chromosome 17 probe ratio > 2.2). Thirty-two tumors were amplified with dual color SISH while negative with mono color SISH, and two were amplified with mono color SISH but were negative for HER2 amplification with dual color SISH. These 34 tumors were thus considered to be discordant between mono color and dual color SISH. Results from the 34 discordant tumors were revised. At this repeated assessment, 11 tumors initially scored as HER2 amplified with dual color SISH were scored as negative for amplification, eight tumors were scored equivocal for amplification and 11 tumors were again scored as HER2 amplified. At repeated assessment of mono color SISH results, two tumors that were initially scored as negative for amplification were scored as HER2 amplified and one tumor initially scored as positive was scored as HER2 negative. After this revision, the number of discordant results was reduced to seven. All these tumors were amplified with dual color SISH (ratios were between 2.2 and 2.97) while no amplification was found with mono color SISH. HER2 gene copy numbers for these tumors with mono color SISH were two (one case), three (five cases) or four (one case). We compared the IHC results for these discordant cases, and only one case showed 3+ staining for at least one of the antibodies used when dual color SISH showed HER2 gene amplification, but mono color SISH showed no HER2 gene amplification. We decided to use the revised mono color SISH results to determine HER2 gene amplification for this HER2 TMA assessment. Overall, correlation between these two SISH methods when considering amplified and non-amplified tumors was very high after revision (Table 1) (κ = 0.952).

**Table 1:** Mono color SISH versus dual color SISH.

Dual color SISH				
		**Non-Amplified (ratio < 1,8)**	**Equivocal (1,8 < ratio < 2,2)**	**Amplified (ratio > 2,2)**

**Mono color SISH**	Non-Amplified (< 6 copy numbers)	841	24	7
	Amplified (> 6 copy numbers)	0	2	88

SISH, silver *in situ *hydridization.

### Comparison of the 4B5, SP3 antibodies with the Herceptest

For comparing the performance of the three different HER2 antibodies in detecting HER2 protein expression on TMAs, only those cases were evaluated that had both informative IHC and mono color SISH scores, which resulted in over 1,000 interpretable cases for each (Table 2). Performance of SP3, 4B5 and Herceptest antibodies was evaluated based on the number of 'false negative' results (0,1+ IHC score, positive for HER2 amplification with mono color SISH) and 'false-positive' results (3+ IHC score, negative for amplification with mono color SISH). Both SP3 and 4B5 showed three false-negative results (0.28% and 0.29%), while Herceptest showed 13 false negative results (1.2%). The number of false-positive results was comparable (four tumors with SP3, six with 4B5 and five with Herceptest). With cases that were scored 3+ on IHC, positive predictive values for the 4B5 and SP3 antibodies and the Herceptest were also comparable (93%, 95% and 93%, respectively (Table 2). Concordance in staining between antibodies was highest between the 4B5 and SP3 antibodies (κ = 0.770), compared to 4B5 and Herceptest (κ = 0.707) and SP3 and Herceptest (κ = 0.768) (Table 3).

**Table 2:** 4B5, SP3, Herceptest and mono color SISH.

4B5	HER2 amplification	no HER2 amplication	SP3	HER2 amplification	no HER2 amplification	Herceptest	HER2 amplification	no HER2 amplification
0,1+	3	907	0,1+	3	924	0,1+	13	951
2+	8	46	2+	13	36	2+	11	18
3+	75	6	3+	69	4	3+	65	5
Total	86	959		85	964		89	974

HER2, human epidermal growth factor receptor 2; SISH, silver *in situ *hybridization.

**Table 3:** SP3, 4B5 and Herceptest.

		SP3					Herceptest					Herceptest		
								
		0,1+	2+	3+			0,1+	2+	3+			0,1+	2+	3+
4B5	0,1+	911	15	0	4B5	0,1+	920	7	0	SP3	0,1+	930	9	0
	2+	30	26	1		2+	42	15	0		2+	27	18	2
	3+	1	7	72		3+	7	8	68		3+	6	3	66
Total		942	48	73	Total		969	30	68	Total		963	30	68

### TMA results

For 1,000 (82.6%) of all 1,210 cases, complete results were obtained with 4B5, SP3, Herceptest and mono color SISH. In 847 tumors of these cases (84.7%) no HER2 protein overexpression was found with SP3, 4B5, Herceptest (0 or 1+) or mono color SISH (HER2 copy number < 6). Sixty tumors showed 3+ staining with all three antibodies and HER2 gene amplification (6.0%). For the remaining 93 complete cases, the final TMA HER2 status was determined as follows: 65 tumors (6.5%) showed 0, 1+ or 2+ IHC scores and were negative for HER2 gene amplification, and were thus judged to be HER2 negative. Twelve tumors (1.2%) showed 3+ or 2+ IHC scores and were positive for HER2 gene amplification and were thus judged to be HER2 positive. For 16 cases (1.6%), there was a true discordance between at least one of the antibodies mono color SISH result (for example, 0/1+ IHC and HER2 gene amplification; 3+ IHC and no HER2 gene amplification).

The most frequent discordant result between TMA scores was a false-negative result with the Herceptest (IHC score of 0/1+ with Herceptest and 2/3+ with SP3 and 4B5 and positive SISH) which occurred in eight cases. Tumors stained with Herceptest frequently displayed prominent cytoplasmic and moderate but incomplete membranous staining (1+), while 4B5 and SP3 antibodies displayed complete membranous staining. There were four cases for which all three antibodies showed 3+ staining on TMA, while mono color SISH did not detect HER2 gene amplification. Other discordant results include 0/1+ staining with all three antibodies while mono color SISH showed HER2 gene amplification (three cases) and 3+staining with 4B5 and Herceptest while SP3 and mono color SISH were negative (one case). When Herceptest results were omitted, the number of interpretable cases for 4B5, SP3 and mono color SISH increased to 1,020 and the number of discordant results was reduced to eight (0.8%). In total, 932 tumors were HER2 negative and 80 tumors were HER2 positive when determined with these three methods (Table 4). Therefore, the final HER2 status on TMA was determined with SP3, 4B5 and mono color SISH.

**Table 4:** HER2 status determined on TMA (SP3, 4B5 and mono color SISH)

Total complete results	1,020 (84.3%)
Negative	932 (91.4%)
Positive	80 (7.8%)
Discordant results	8 (0.8%)
Incomplete results	190 (15.7%)
Total number of cases	1210 (100%)

HER2, human epidermal growth factor receptor 2; SISH, silver *in situ *hybridization; TMA, tissue micro-array.

### Comparison of local result with TMA result

For determining the concordance between local testing result and the TMA retesting, we compared the HER2 scores listed in archival pathology reports from the local centers with the final HER2 status determined by TMA slides stained with SP3, 4B5 and mono color SISH. Mono color SISH was used to determine HER2 status on the TMA when IHC and mono color SISH were discordant. For 1,008 tumors, complete information was available. We found discrepancies between the TMA result and the result listed in the pathology report in 30 cases. In order to evaluate these cases further, all local testing material was revised and full-sized slides were stained with SP3, 4B5 and mono color SISH. When the original HER2 gene amplification was initially determined on core needle biopsy, for our study these stains were repeated on both biopsy and resected tumors.

Ten tumors with discordant results between TMA and local result were found to be concordant after staining full-sized slides. HER2 expression and gene amplification was likely to be heterogeneously present in these tumors, and the use of TMAs had led to a sampling error. Thus, the total amount of discordant results between local testing and this TMA testing was reduced to 20. Total percentage of concordance for all pooled cases from different centers was 98.0% (range 94.4% to 99.4%). Sensitivity and specificity for all centers combined was 98.7% and 99.3%, respectively (Table 5).

**Table 5:** Performance of participating HER2 testing centers.

Hospital	Total	False-negative	False-positive	Concordance	Sensitivity (%)	Specificity (%)
1	144	3 (2.1%)	2 (1.4%)	139 (96.5%	98.6	97.9
2	266	0 (0.0%)	2 (0.8%)	264 (99.2%)	99.2	100.0
3	158	1 (0.6%)	0 (0.6%)	156 (98.7%)	100.0	99.4
4	181	2 (1.1%)	2 (1.1%)	177 (97.8%)	98.9	98.9
5	90	0 (0.0%)	5 (5.6%)	85 (94.4%)	94.4	100.0
6	169	1 (0.6%)	2 (1.2%)	166 (98.2%)	98.8	100.0
Total	1,008	7 (0.7%)	13 (1.3%)	988 (98.0%)	98.7	99.3

HER2, human epidermal growth factor receptor 2.

Of the 20 discordant cases, 13 were scored as HER2 positive in local centers but were negative in our TMA retesting (1.3% of the total of 1,008 cases) (Table 6). Another seven cases were scored as HER2 negative in local centers, but were positive upon retesting (0.7%) (Table 7). For the 13 local positive results which were negative with TMA retesting, seven of these had a local 3+ result, and six cases were found positive in local laboratories due to 2+ IHC and amplification by ISH methodology. The slides used for the original diagnosis at the local centers were revised by a panel of pathologists in order to assess local observer reliability. Four tumors that were scored as 3+ in local hospitals were scored as 2+ by the revision panel and thus reflect observer inaccuracy. All local 2+ tumors were correctly identified as 2+ on IHC. For two tumors, the local ISH result was decided to be non-amplified by the revision panel, while the local observer scored the tumor as HER2 amplified.

**Table 6:** Local positive, TMA negative results (*n *= 13)

**No. of cases**	**Local result**	**Revision local result**	**TMA result**	**Full-sized 4B5, SP3, monocolor-SISH result**	**Conclusion**
		
4	2+, ISH+	2+, ISH+	No amplification	2+, 2+, no amplification	Local -ISH procedure unreliable
1	3+	3+	No amplification	3+, 3+ no amplification	Overexpression without amplification
2	3+	3+	No amplification	2+, 2+ no amplification	Local IHC procedure unreliable
1	3+	2-3+	No amplification	2-3+, 2+, no amplification	Local IHC scoring unreliable
3	3+	2+	No amplification	1-2+, 1-2+, no amplification	Local IHC scoring unreliable
2	2+, ISH+	2+, ISH -	No amplification	2+, 2 no amplification	Local -ISH scoring unreliable

IHC, immunohistochemistry; ISH, *in situ *hybridization; SISH silver *in situ *hybridization; TMA, tissue micro-array.

**Table 7:** Local negative, TMA positive results (number = 7).

No. of cases	Local result	Revision local result	TMA result	Full-sized 4B5, SP3, monocolor-SISH result	Conclusion
3	0+	0+	Amplification	2+, amplification	Local IHC procedure unreliable
3	0+	0+	Amplification	3+, amplification	Local IHC procedure unreliable
1	0+	1-2+	Amplification	2+, amplification	Local IHC scoring unreliable

**Figure 4:**
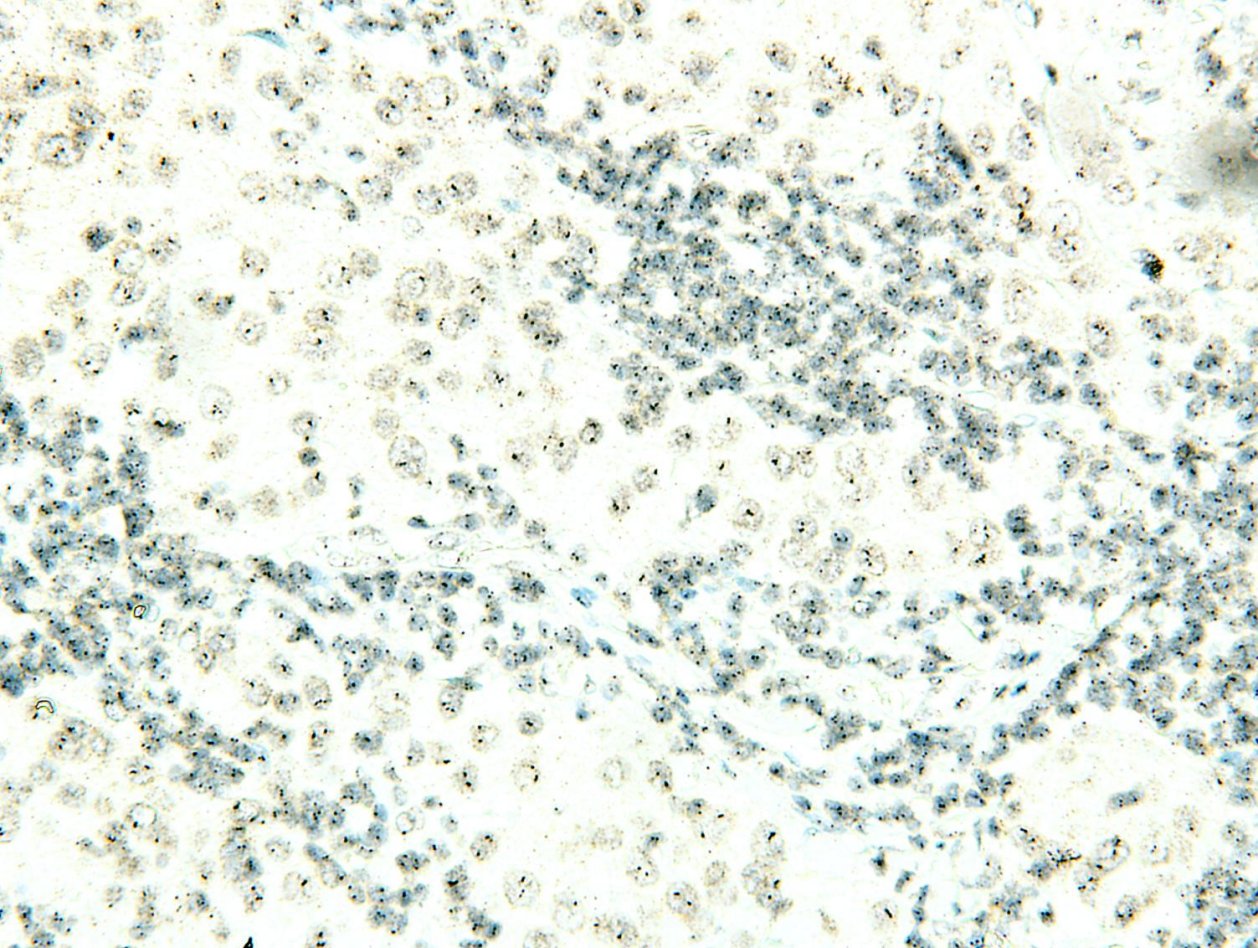
**Tumor that displayed HER2 protein overexpression in the absence of gene amplification (mono color SISH negative)**. HER2, human epidermal growth factor receptor 2; SISH, silver *in situ *hybridization.

**Figure 5:**
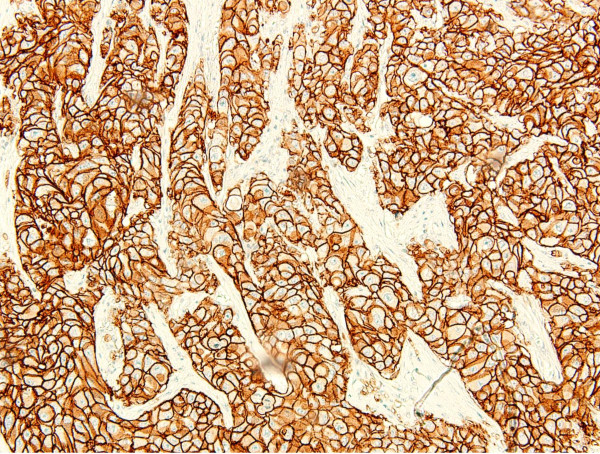
**Tumor that displayed HER2 protein overexpression in the absence of gene amplification (3+, SP3 antibody)**. HER2, human epidermal growth factor receptor 2.

For the remaining discordant cases, whole tumor slides stained with SP3, 4B5 and mono color SISH were evaluated in order to assess the reason for the discordant result. For the 2+, ISH amplified results, complete membranous staining on IHC could be reproduced with the SP3 and 4B5 antibodies in all cases. The local ISH results showed low level HER2 gene amplification (six to ten) in four of six tumors and high level HER2 gene amplification (> 10 copies) in two of six tumors, but all were negative for amplification determined with mono color SISH on both TMA and subsequent testing of the whole tumor. These data indicate that the local IHC result was reliable, but the reason for these results was false-positive local ISH due to the ISH procedure. For the remaining three local 3+ results, 3+ staining could not be reproduced with SP3 and/or 4B5 antibodies on TMA for two tumors and these were both negative for gene amplification on mono color SISH on TMA and on full-sized slides. For these tumors, the reason for discordance was the local IHC procedure leading to false 3+ results. The remaining tumor was 3+ on local slides and was also 3+ on SP3 and 4B5 stained slides, while the mono color SISH result was non-amplified (two to three copy numbers). This tumor thus likely represents a case of protein overexpression without gene amplification (Figure 4 and 5).

For all false-negative results, all seven local slides were revised to assess observer performance. One of these tumors was mistakenly interpreted as negative on IHC while the tumor showed 2+ membranous staining in parts of the tumor. The resulting six tumors were negative, but did show 2+ or 3+ results with 4B5 and SP3 stained slides, and amplification with mono color SISH. This reflects an inaccurate local IHC procedure, which resulted in false-negative results.

## Discussion

Breast cancer is the most frequent form of cancer in women with an incidence of 421,000 new cases in Europe in 2008 [18]. HER2 testing is considered the standard of care for all breast cancer patients as this can determine neoadjuvant, adjuvant and metastatic treatment. Due to the increasing demand for HER2 testing, reliable HER2 testing methods are necessary. Earliest reports into the concordance and reliability of local HER2 tests revealed a significant amount of discordance [15,16]. In order to improve the standardization of HER2 testing, external quality controls have been developed in order to compare HER2 testing outcomes between laboratories which can ensure that HER2 testing leads to the same results irrespective of which laboratory performs the HER2 test. Dowsett *et al*. presented the results from an international ring study that sent 20 blank slides from a selection of HER2 amplified and HER2 non-amplified breast cancer specimens from one center to another, with each center performing both IHC and FISH according to local methods [19]. Other studies have created HER2 testing controls, specifically designed to be used for the purpose of quality control schemes. Rhodes *et al*. sent one slide containing four cell line blocks with graded and constant HER2 protein levels, two of these cell lines were previously diagnosed as HER2 non-amplified and two were HER2-amplified [20]. Slides were sent to 90 laboratories in 21 countries, which all used their own methods for detecting HER2 protein expression and HER2 gene amplification [21]. These approaches allow the participation of high numbers of HER2 testing laboratories and allow for the identification of methods that lead to false-positive and false-negative results. The downside of these methods is that the number of tumors or cell lines tested in these studies was limited. The use of TMAs enables the use of hundreds of different tumors in the same procedure by analyzing one single TMA slide. The obvious downside to this method is that using TMAs enables the analysis of only limited amounts of tissue per tumor. However, the analysis of two cores for HER2 status has been shown to correlate with the whole-tumor sections in more than 95% of cases [22]. We hypothesized that TMAs might be used in retesting a high number of previously HER2-tested breast cancers. For this approach, local testing centers would send a number of 100+ HER2 tested invasive breast cancers which would all be included in the TMAs. The HER2 TMA result would be compared to the locally determined HER2 status in order to assess the reliability. Because using TMAs introduces the possibility of sampling error due to tissue heterogeneity, discordant results would be decided by testing whole tumor sections and revision of the local slides that were used in the initial HER2 testing. This method allows the assessment of the HER2 testing performance in a relatively high number of tumors and the identification of the local laboratory failure (either due to HER2 staining evaluation or procedure). Using this approach has the additional advantage that it uses locally tested and treated tumors, possibly providing a more reliable evaluation of HER2 testing performance than when artificial cell lines are used. Because the retesting results would be available to the local testing center, this might also lead to information that might benefit patients in future follow-up. In order to investigate the feasibility of TMAs for HER2 testing quality assessment, we have retested HER2 status in approximately 1,200 recently diagnosed breast carcinomas from patients that were tested using various HER2 testing reagents in six different pathology laboratories in the Netherlands. Because this was a pilot study, the HER2 testing TMAs were stained with three different antibodies and mono color SISH and dual color SISH in order to ascertain optimal HER2 testing methods for the purpose of this TMA evaluation. Mono color SISH and SP3 and 4B5 antibodies were used to determine the final HER2 status for the tumors on the TMA.

Based on the publications of ASCO/CAP, we hypothesized that severe discrepancies between local results and the TMA test would be found. However, in contrast to our hypothesis, our results show unexpectedly high concordance in each institution, indicating high reproducibility and reliability of HER2 testing in these laboratories. All these centers are hospitals with relatively high-volumes of HER2 testing which increases the reliability of testing results [17]. False negative test results were identified in 0.7% of the cases and false positive test results in only 1.3% of the cases. Reasons for these 20 discordant cases were variable; four cases were due to local inaccurate ISH assay procedures (20.0%), two discordant cases were due to inaccurate scoring of ISH assays (10.0%), eight were due to local inaccurate IHC procedures (40.0%) and five were due to inaccurate scoring of IHC assays (25.0%). The remaining discordant case was a tumor with 3+ scores on local IHC which tested 3+ with SP3, 4B5 and Herceptest, but was negative with mono color SISH. Although several studies have reported ISH negative tumors with 3+ IHC results [23], these were generally all tested with a single antibody, meaning that these discordant results could reflect technical issues associated with HER2 testing. Since this tumor has positive results with multiple antibodies, this tumor is likely to indeed have overexpression of the HER2 protein in the absence of gene amplification. Overexpression of the HER2 protein without amplification at the genomic level has been described previously to be rare [24].

FISH is traditionally considered the gold standard for assessing HER2 gene amplification. Concordance between locally and centrally performed FISH assays have been shown to be higher compared to IHC [16]. Other probe-based assays have been approved for clinical use, notably CISH and SISH, which both use light-microscopy. The advantage of light microscopy is that this allows simultaneous evaluation of the invasive tumor component and HER2 amplification. We have used mono color SISH in our study as the gold standard. It has been previously demonstrated that this method has very high concordance with FISH [25]. We compared the concordance between mono color and dual color SISH. Dual color SISH is only considered amplified when the HER2 to chromosome 17 probe ratio exceeds 2.2. Importantly, loss of the chromosome 17 probe binding region can lead to a falsely elevated HER2 to chromosome 17 ratio which is likely unrelated to HER2 status. The recommendation is therefore not to qualify any tumor as HER2 amplified unless there are at least four HER2 copy numbers, regardless of the HER2/chromosome 17 ratio. Secondly, for tumors with polysomy 17 and concomitant HER2 gene amplification, this ratio will not exceed 2.2 and these tumors will thus be considered HER2 non-amplified. Since mono color SISH only includes one probe for the HER2 gene, some polysomy 17 tumors will be considered HER2 amplified with this method. Mono color SISH results are considered amplified when the number of HER2 copies exceeds six. Some authors have recommended that in the cases of four to six HER2 spots on mono color SISH, dual hybridization assays should be performed which might lead to identification of some HER2 amplified tumors [26]. The concordance and correlation between these mono color and dual color SISH methods was high in our study We decided to use mono color SISH for determining HER2 gene status for this TMA assessment, since this seemed to correlate better to IHC results for the few discordant cases.

We compared the characteristics of two monoclonal rabbit antibodies, 4B5 and SP3, with the Herceptest, for the assessment of HER2 protein expression on the TMAs. Herceptest displayed the lowest sensitivity in our study, as this antibody had the highest number of tumors that tested 0 or 1+, but were positive for HER2 amplification on mono color SISH. This is in accordance with another study comparing A0485, CB11, TAB250 and the Herceptest, in which the Herceptest was found to have the lowest sensitivity [27].

The 4B5 is a recently developed rabbit monoclonal antibody which has been previously compared to the CB11 antibody, the mouse monoclonal antibody that is used in the FDA-approved PATHWAY kit (Ventana). SP3 is another monoclonal rabbit antibody, which has also been compared to the CB11 antibody [28] and A0485, 4D5 CM-CB11 and Herceptest [29]. SP3 was found to have a higher sensitivity than the Herceptest, which is in accordance with our study.

## Conclusions

In conclusion, we have shown that TMAs can be used to evaluate the quality of HER2 testing in pathology laboratories. This provides a reliable evaluation of HER2 testing performance. In the first assessment performed in this way, we found HER2 testing sensitivity to be 98.7% and specificity to be 99.3%. As we have now established this TMA-based evaluation in combination with full-sized slides for discordant cases, we will also offer this to other laboratories.

## Abbreviations

ASCO/CAP: American Society of Clinical Oncology/College of American Pathologists; CISH: chromogenic *in situ *hybridization; FISH: fluorescence *in situ *hybridization; H & E: haematoxylin and eosin; HER2: human epidermal growth factor receptor 2; IHC: immunohistochemistry; ISH: *in situ *hybridization; NSABP: National Surgical Adjuvant Breast and Bowel Project; SISH: silver *in situ *hybridization; TMA: tissue micro-array.

## Competing interests

All participating hospitals have received funding for this study from Hoffman-La Roche. The following additional possible financial conflicts of interests have been declared: TJA Dekker received lecture honoraria from Hoffman-La Roche. S Ter Borg received lecture honoraria from Hoffman-La Roche. JE Boers has received travel reimbursements, lecture honoraria and research funding from Hofman-La Roche. MJ van de Vijver has received research funding, lecture honoraria and is member of the pathology advisory board from Hofman-La Roche. E Schuuring has received travel reimbursements, lecture honoraria and occasional honoraria for advisory board member for Hoffman-La Roche, and received lecture honoraria from Abbott (all transferred to UMCG account).

## Authors' contributions

TJAD, STB and GKJH participated in data collection, interpretation and analysis. SM, JW, JEB, ES, JB and VTHBMS participated in the design of the study, included tumor material in the study and scored slides. WEM and JRK participated in writing of the manuscript and data interpretation. MJvdV coordinated the study, participated in its design, data interpretation and writing of the manuscript. All authors have read and approved the manuscript.
